# Supplementary material for: How to Improve the Reliability of Aperiodic Parameter Estimates in M/EEG: A Method Comparison and Recommendations for Best Practices
Source: bioRxiv. 2025 Nov 11:2025.11.10.687541. Preprint. [Version 1] doi: 10.1101/2025.11.10.687541 (PMC12642415; doi:10.1101/2025.11.10.687541)
Supplement: Supplement 1 [file media-1.pdf]

Figure S1 demonstrates the robustness of the censored regression approach to changes in the width of the censored region. To illustrate that our censored regression approach sacrifices little useful information for the estimation of aperiodic parameters through the censoring step, we simulated 500 power spectra in log–log space as a linear function of frequency (2-33 Hz), with a slope of -1.2 and an intercept of 1.0. An oscillatory peak with a Gaussian shape centered around 10 Hz was added to each spectrum, along with random Gaussian noise to model measurement variability. Slopes were then estimated using four approaches for each spectrum:

1. full regression across the entire 2–33 Hz range, labelled "full" in the figure below
2. censored regression with 6–16 Hz excluded (as in the main manuscript), labelled "truncated" in the figure below
3. a more aggressive censoring approach, excluding 4–20 Hz, labelled "aggressive" in the figure below
4. extreme censoring, using only the first and last frequencies (i.e., only 2 points), labelled "bookend" in the figure below

The dashed line indicates the ground truth slope value.

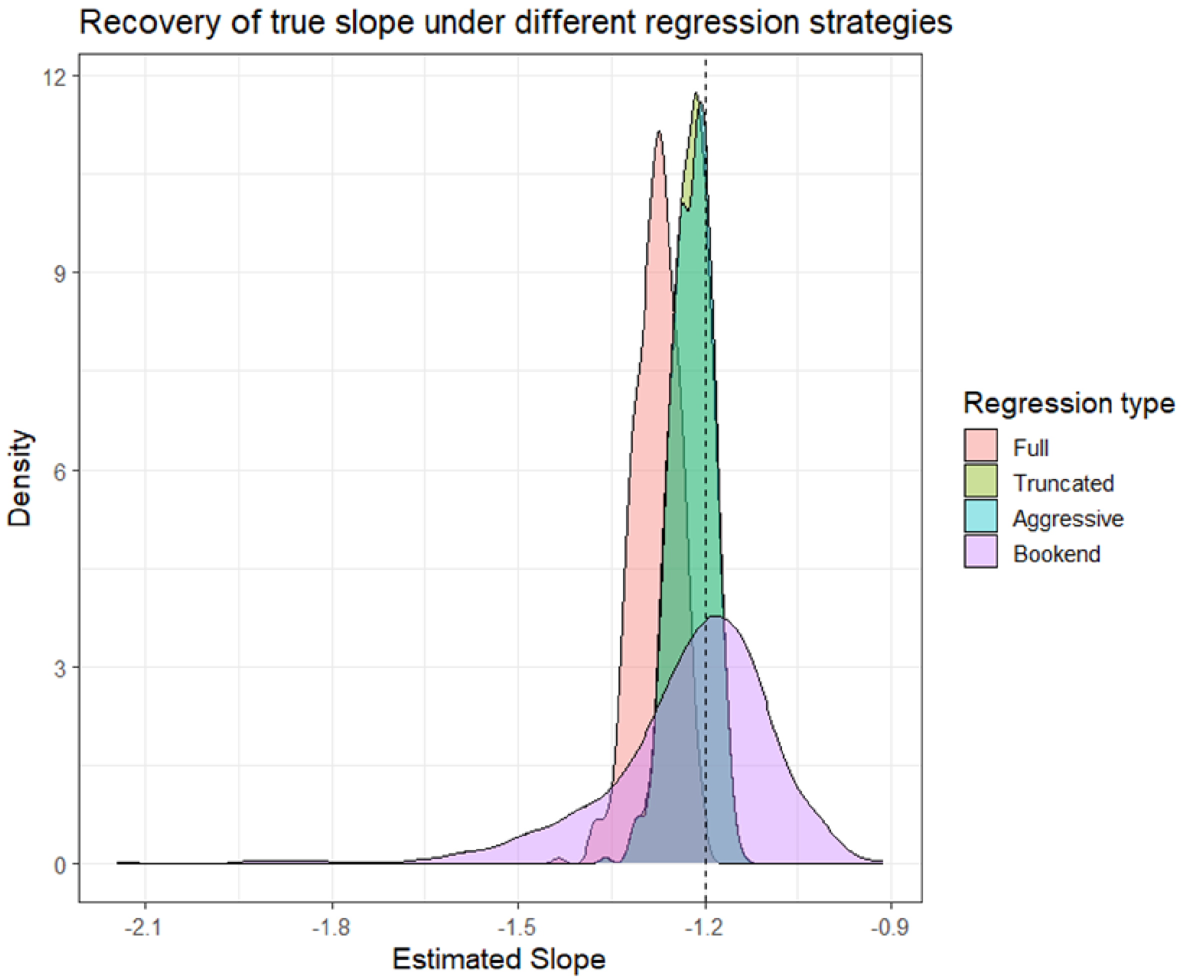

These findings clearly suggest that 1) full regression (red) misestimates the ground truth parameter, in line with the conclusions of our manuscript, 2) the two reasonable censoring approaches lead to practically indistinguishable estimates even though they censor different numbers of points, including far more points not contaminated by the oscillatory peak in the aggressive (blue) compared to the regular truncated (green) approach, and 3) even an extreme censoring approach (purple), that leaves only two points for slope estimation, leads to estimates centered roughly around the ground truth value, albeit with larger spread than any other approaches, reflecting this method's sensitivity to noise.

Table S1.

Average slope and intercept values across participants and electrode for each estimation approach after outlier removal

Spectral Decomposition	Aperiodic Estimation	Slope	MeanSlope	SDSlope Min	Slope	MaxIntercept Mean	Intercept SD	Intercept Min	Intercept Max
**Resting State Dataset**									
**N = 61, EEG Electrodes = 59. mean epochs = 45.45**									
FFT	fooof0	-1.06	0.53	-5.19	0.00	0.89	0.75	-1.39	6.81
FFT	fooof1	-1.11	0.66	-6.37	0.00	0.71	0.82	-1.89	7.11
FFT	fooof3	-1.20	0.65	-6.38	0.00	0.69	0.83	-2.10	7.14
FFT	reg-full	-1.06	0.53	-5.9	0.00	0.89	0.75	-1.39	6.81
FFT	reg-censor	-1.00	0.51	-5.06	0.00	0.78	0.71	-1.58	6.61
Welch	fooof0	-1.04	0.57	-5.68	0.00	0.82	0.76	-1.47	7.41
Welch	fooof1	-1.10	0.68	-6.21	0.00	0.64	0.81	-1.83	7.49
Welch	fooof3	-1.19	0.67	-6.91	0.00	0.62	0.81	-2.07	7.59
Welch	reg-full	-1.05	0.57	-5.36	0.00	0.82	0.76	-1.47	7.41
Welch	reg-censor	-0.98	0.51	-5.06	0.00	0.71	0.71	-1.58	7.20
**Stop-Signal Task Dataset**									
**N = 34, EEG Electrodes = 32, mean epoch range 607-779**									
FFT	fooof0	-1.34	0.37	-2.49	-0.23	1.13	0.43	-0.26	2.55
FFT	fooof1	-1.35	0.55	-3.19	-0.03	0.86	0.63	-1.04	2.74
FFT	fooof3	-1.41	0.57	-3.21	-0.02	0.83	0.65	-1.14	2.71
FFT	reg-full	-1.34	0.37	-2.50	-0.23	1.13	0.43	-0.26	2.55
FFT	reg-censor	-1.28	0.37	-2.43	-0.15	1.01	0.44	-0.50	2.40
Welch	fooof0	-1.39	0.34	-2.49	-0.35	1.34	0.40	0.10	2.72
Welch	fooof1	-1.37	0.39	-2.62	-0.22	1.14	0.43	-0.28	2.57
Welch	fooof3	-1.42	0.38	-2.63	-0.25	1.14	0.43	-0.29	2.53
Welch	reg-full	-1.39	0.34	-2.49	-0.35	1.34	0.40	0.10	2.72
Welch	reg-censor	-1.33	0.33	-2.40	-0.29	1.23	0.39	-0.04	2.56

Note. Data were averaged across epochs, channels, and participants for each combination of Spectral Decomposition and Aperiodic Estimation. *FFT*, fast Fourier transform; *Welch*, Welch's method; *fooof0, fooof1,* and *fooof3, fooof*-derived slope estimates with the corresponding number of modeled peaks; *reg-full*, full regression approach; *reg-censor*, censored regression approach.

Table S2. Frequency of Improbable Values. **All Positive Slopes**, Bootstrap Analyses Ranked Results for Both Datasets.

Spectral Decomposition	Aperiodic Estimation	Probability of being the Best	Mean Rank (smaller is better)
Resting State Dataset – Eyes Open			
FFT	reg-censor	98.26	1.0350
FFT	fooof0	1.76	2.4825
FFT	reg-full	1.76	2.4825
Welch	reg-censor	0.00	4.3350
Welch	fooof0	0.00	5.3379
Welch	reg-full	0.00	5.3379
FFT	fooof1	0.00	7.2815
FFT	fooof3	0.00	7.7077
Welch	fooof3	0.00	9.0000
Welch	fooof1	0.00	10.0000
Resting State Dataset – Eyes Closed			
FFT	fooof0	100	1.5000
FFT	reg-full	100	1.5000
FFT	reg-censor	0	3.0012
Welch	fooof0	0	4.4994
Welch	reg-censor	0	4.4994
Welch	reg-full	0	6.0000
FFT	fooof3	0	7.0000
FFT	fooof1	0	8.0026
Welch	fooof3	0	8.9974
Welch	fooof1	0	10.0000
Stop-Signal Task Dataset			
Welch	fooof3	99.96	1.0004
Welch	reg-censor	0.04	1.9996
Welch	fooof1	0	3.0044
Welch	fooof0	0	4.4978
Welch	reg-full	0	4.4978
FFT	reg-censor	0	6.0034
FFT	fooof0	0	7.0039
FFT	reg-full	0	7.9927
FFT	fooof1	0	9.0000
FFT	fooof3	0	10.000

Table S3. Frequency of Improbable Values. **Positive Slopes Not Explained**, Bootstrap Analyses Ranked Results for Both Datasets

Spectral Decomposition	Aperiodic Estimation	Probability of being the Best	Mean Rank (smaller is better)
Resting State Dataset – Eyes Open			
FFT	reg-censor	72.20	1.3193
Welch	reg-censor	36.06	1.6807
FFT	fooof0	0.00	3.5000
FFT	reg-full	0.00	3.5000
Welch	fooof0	0.00	5.5000
Welch	reg-full	0.00	5.5000
Welch	fooof3	0.00	7.7402
FFT	fooof1	0.00	8.1279
Welch	fooof1	0.00	8.1319
FFT	fooof3	0.00	10.0000
Resting State Dataset – Eyes Closed			
FFT	reg-censor	90.58	1.0950
Welch	reg-censor	9.58	1.9050
FFT	fooof0	0.00	3.5000
FFT	reg-full	0.00	3.5000
Welch	fooof0	0.00	5.5000
Welch	reg-full	0.00	5.5000
Welch	fooof3	0.00	7.0006
FFT	fooof3	0.00	8.0246
FFT	fooof1	0.00	9.1058
Welch	fooof1	0.00	9.8690
Stop-Signal Task Dataset			
FFT	reg-censor	100	1.5000
Welch	reg-censor	100	1.5000
Welch	fooof3	0	3.0004
Welch	fooof0	0	4.5314
Welch	reg-full	0	4.5314
FFT	reg-full	0	6.2835
FFT	fooof0	0	6.9275
Welch	fooof1	0	7.7258
FFT	fooof1	0	9.0000
FFT	fooof3	0	10.000

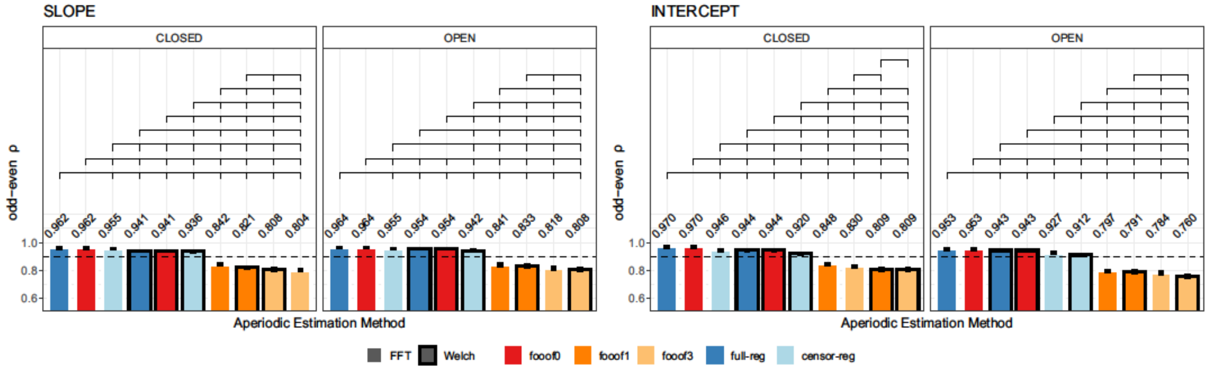

Figure S2. Internal consistency (odd-even reliability) after removing positive slopes for slope (left) and intercept (right) across aperiodic estimation methods, shown separately for eyes-closed and eyes-open during resting state. Within each subplot, bars are ordered from highest to lowest reliability. Brackets above the bars indicate statistically significant differences ($p < .05$ using permutation testing). Error bars reflect within-subject standard errors of the mean (SEM), computed using Morey's (2008) correction for repeated-measures designs. The horizontal dashed line represents a reliability threshold of .90, considered acceptable.

Table S4. **Exponent** Odd-Even Reliability - Resting State Dataset - Bootstrap Analyses Results

Spectral Decomposition	Aperiodic Estimation	Probability of being Best	Mean Rank (smaller is better)
Eyes Open – Positive Slopes Retained			
FFT	fooof0	100	1.5000
FFT	reg-full	100	1.5000
Welch	fooof0	0	3.5093
Welch	reg-full	0	3.5093
FFT	reg-censor	0	4.9814
Welch	reg-censor	0	6.0000
Welch	fooof1	0	7.4881
FFT	fooof1	0	7.5119
FFT	fooof3	0	9.0000
Welch	fooof3	0	10.0000
Eyes Closed - Positive Slopes Retained			
FFT	fooof0	100	1.5000
FFT	reg-full	100	1.5000
FFT	reg-censor	0	3.0000
Welch	fooof0	0	4.5002
Welch	reg-full	0	4.5002
Welch	reg-censor	0	5.9996
FFT	fooof1	0	7.2865
Welch	fooof1	0	7.7135
FFT	fooof3	0	9.3836
Welch	fooof3	0	9.6164
Eyes Open – Positive Slopes Removed			
FFT	fooof0	100.0	1.1795
FFT	reg-full	35.9	1.8205
FFT	reg-censor	0.0	3.4504
Welch	fooof0	0.0	4.2748
Welch	reg-full	0.0	4.2748
Welch	reg-censor	0.0	6.0000
FFT	fooof1	0.0	7.0314
Welch	fooof1	0.0	7.9711
FFT	fooof3	0.0	9.0265
Welch	fooof3	0.0	9.9710
Eyes Closed – Positive Slopes Removed			
FFT	fooof0	100	1.5000
FFT	reg-full	100	1.5000
FFT	reg-censor	0	3.0000
Welch	fooof0	0	4.5000
Welch	reg-full	0	4.5000
Welch	reg-censor	0	6.0000
FFT	fooof1	0	7.0000
Welch	fooof1	0	8.0006
Welch	fooof3	0	9.1758
FFT	fooof3	0	9.8236

Table S5. **Offset** Odd-Even Reliability - Resting State Dataset – Bootstrap Analyses Results

Spectral Decomposition	Aperiodic Estimation	Probability of being Best	Mean Rank (smaller is better)
Eyes Open – Positive Slopes Retained			
FFT	fooof0	99.98	1.5004
FFT	reg-full	99.98	1.5004
Welch	fooof0	0.02	3.4996
Welch	reg-full	0.02	3.4996
FFT	reg-censor	0.00	5.0000
Welch	reg-censor	0.00	6.0000
Welch	fooof1	0.00	7.2848
FFT	fooof1	0.00	7.7325
FFT	fooof3	0.00	8.9841
Welch	fooof3	0.00	9.9986
Eyes Closed - Positive Slopes Retained			
FFT	fooof0	100.00	1.1799
FFT	reg-full	35.98	1.8201
Welch	fooof0	0.00	3.7117
Welch	reg-full	0.00	3.7117
FFT	reg-censor	0.00	4.5766
Welch	reg-censor	0.00	6.0000
FFT	fooof1	0.00	7.0083
FFT	fooof3	0.00	7.9927
Welch	fooof1	0.00	9.2984
Welch	fooof3	0.00	9.7006
Eyes Open – Positive Slopes Removed			
FFT	fooof0	100	1.5000
FFT	reg-full	100	1.5000
Welch	fooof0	0	3.5000
Welch	reg-full	0	3.5000
FFT	reg-censor	0	5.0000
Welch	reg-censor	0	6.0000
FFT	fooof1	0	7.1231
Welch	fooof1	0	8.0036
FFT	fooof3	0	8.8733
Welch	fooof3	0	10.0000
Eyes Closed– Positive Slopes Removed			
FFT	fooof0	100	1.5000
FFT	reg-full	100	1.5000
FFT	reg-censor	0	3.3426
Welch	fooof0	0	4.3287
Welch	reg-full	0	4.3287
Welch	reg-censor	0	6.0000
FFT	fooof1	0	7.0000
FFT	fooof3	0	8.0008
Welch	fooof1	0	9.4631
Welch	fooof3	0	9.5361

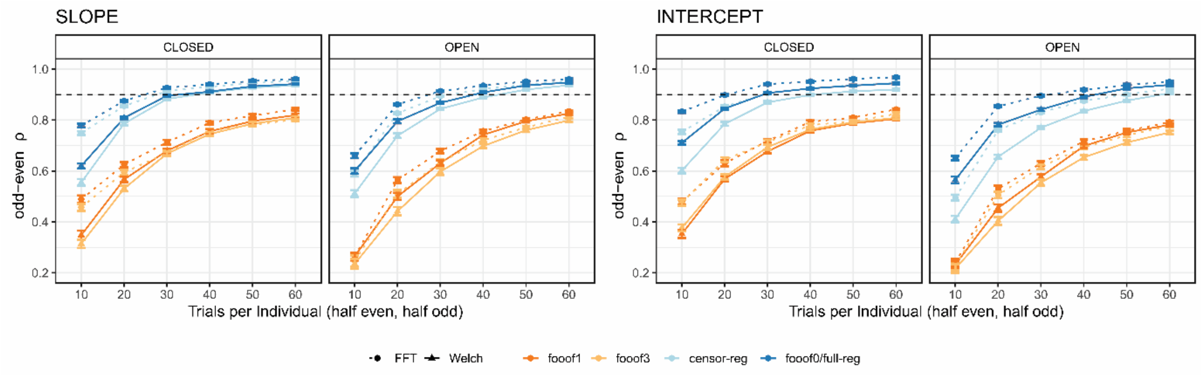

Figure S3. Internal consistency (odd-even reliability) as a function of trial count after removing positive slopes for slope (left) and intercept (right) across aperiodic estimation methods, shown separately for eyes-closed and eyes-open resting state. The horizontal dashed line represents a reliability threshold of .90, considered acceptable.

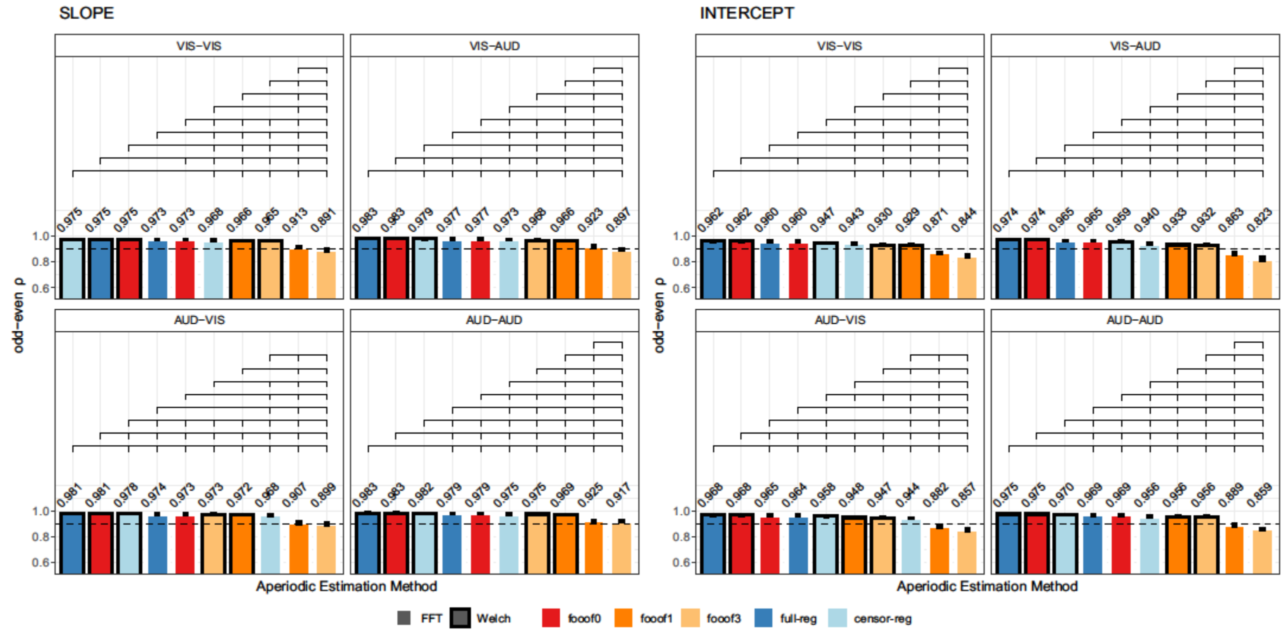

Figure S4. Internal consistency (odd-even reliability) after removing positive slopes for slope (left) and intercept (right) across aperiodic estimation methods, shown separately for each version of the stop-signal task. Within each subplot, bars are ordered from highest to lowest reliability. Brackets above the bars indicate statistically significant differences ($p < .05$, using permutation testing). Error bars reflect within-subject standard errors of the mean (SEM), computed using Morey's (2008) correction for repeated-measures designs. The horizontal dashed line represents a reliability threshold of .90, considered acceptable.

Table S6. **Exponent, Positive Slopes Retained,** Odd-Even Reliability - Stop Signal Dataset – Bootstrap Analyses Results

Spectral Decomposition	Aperiodic Estimation	Probability of being the Best	Mean Rank (smaller is better)
Auditory-Auditory			
Welch	fooof0	94.62	1.5575
Welch	reg-full	94.62	1.5575
Welch	reg-censor	6.12	2.8919
FFT	fooof0	0.00	4.2828
FFT	reg-full	0.00	4.2747
Welch	fooof3	0.00	6.3158
FFT	reg-censor	0.00	6.6706
Welch	fooof1	0.00	7.9992
FFT	fooof1	0.00	9.0010
FFT	fooof3	0.00	9.9990
Auditory-Visual			
Welch	fooof0	99.98	1.5002
Welch	reg-full	99.98	1.5002
Welch	reg-censor	0.00	2.9996
FFT	reg-full	0.00	4.7953
FFT	fooof0	0.00	5.2409
Welch	fooof3	0.00	5.6686
Welch	fooof1	0.00	6.3007
FFT	reg-censor	0.00	7.9945
FFT	fooof1	0.00	9.0002
FFT	fooof3	0.00	9.9998
Visual-Auditory			
Welch	fooof0	100	1.5000
Welch	reg-full	100	1.5000
Welch	reg-censor	0	3.0696
FFT	reg-full	0	4.0901
FFT	fooof0	0	4.8403
FFT	reg-censor	0	6.0292
Welch	fooof3	0	7.0775
Welch	fooof1	0	7.8933
FFT	fooof1	0	9.0000
FFT	fooof3	0	10.000
Visual-Visual			
Welch	fooof0	86.84	1.6584
Welch	reg-full	86.84	1.6584
Welch	reg-censor	12.82	2.9563
FFT	reg-full	1.00	4.2311
FFT	fooof0	0.84	4.4966
FFT	reg-censor	0	6.2155
Welch	fooof3	0	7.3543
Welch	fooof1	0	7.4294
FFT	fooof1	0	9.0000
FFT	fooof3	0	10.000

Table S7. **Exponent, Positive Slopes Removed,** Odd-Even Reliability - Stop Signal Dataset – Bootstrap Analyses Results

Spectral Decomposition	Aperiodic Estimation	Probability of being the Best	Mean Rank (smaller is better)
Auditory-Auditory			
Welch	fooof0	94.12	1.5632
Welch	reg-full	94.12	1.5632
Welch	reg-censor	6.76	2.8824
FFT	fooof0	0.00	4.3033
FFT	reg-full	0.00	4.6938
Welch	fooof3	0.00	6.4013
FFT	reg-censor	0.00	6.5928
Welch	fooof1	0.00	8.0000
FFT	fooof1	0.00	9.0008
FFT	fooof3	0.00	9.9992
Auditory-Visual			
Welch	fooof0	99.88	1.5013
Welch	reg-full	99.88	1.5013
Welch	reg-censor	0.14	2.9989
FFT	reg-full	0.00	4.6621
FFT	fooof0	0.00	5.2668
Welch	fooof3	0.00	5.4200
Welch	fooof1	0.00	6.6626
FFT	reg-censor	0.00	7.9870
FFT	fooof1	0.00	9.0088
FFT	fooof3	0.00	9.9912
Visual-Auditory			
Welch	fooof0	100	1.5000
Welch	reg-full	100	1.5000
Welch	reg-censor	0.00	3.0471
FFT	reg-full	0.00	4.0253
FFT	fooof0	0.00	4.9380
FFT	reg-censor	0.00	6.0070
Welch	fooof3	0.00	7.0448
Welch	fooof1	0.00	7.9478
FFT	fooof1	0.00	9.0000
FFT	fooof3	0.00	10.000
Visual-Visual			
Welch	fooof0	82.88	1.6885
Welch	reg-full	82.88	1.6885
Welch	reg-censor	18.04	2.7382
FFT	fooof0	0.20	4.3361
FFT	reg-full	0.40	4.5504
FFT	reg-censor	0.00	6.2802
Welch	fooof1	0.00	7.0448
Welch	fooof3	0.00	7.9478
FFT	fooof1	0.00	9.0000
FFT	fooof3	0.00	10.000

Table S8. **Offset, Positive Slopes Retained,** Odd-Even Reliability - Stop Signal Dataset – Bootstrap Analyses Results

Spectral Decomposition	Aperiodic Estimation	Probability of being the Best	Mean Rank (smaller is better)
Auditory-Auditory			
Welch	fooof0	100	1.5000
Welch	reg-full	100	1.5000
FFT	reg-full	0.00	3.5810
FFT	fooof0	0.00	4.1170
Welch	reg-censor	0.00	4.3020
Welch	fooof3	0.00	6.9289
Welch	fooof1	0.00	6.9555
FFT	reg-censor	0.00	7.1156
FFT	fooof1	0.00	9.0008
FFT	fooof3	0.00	9.9992
Auditory-Visual			
Welch	fooof0	99.74	1.5048
Welch	reg-full	99.74	1.5048
FFT	fooof0	0.00	3.0721
FFT	reg-full	0.00	3.9625
Welch	reg-censor	0.00	3.9558
FFT	fooof1	0.00	6.5625
Welch	fooof3	0.00	6.7858
Welch	reg-censor	0.00	7.6527
FFT	fooof1	0.00	9.0000
FFT	fooof3	0.00	10.000
Visual-Auditory			
Welch	fooof0	100	1.5000
Welch	reg-full	100	1.5000
FFT	fooof0	0	3.3311
FFT	reg-full	0	3.6693
Welch	reg-censor	0	4.9996
FFT	reg-censor	0	6.0182
Welch	fooof1	0	7.4402
Welch	fooof3	0	7.5416
FFT	fooof1	0	9.0000
FFT	fooof3	0	10.000
Visual-Visual			
FFT	fooof0	36.50	2.3172
FFT	reg-full	27.88	2.5034
Welch	fooof0	44.34	2.5897
Welch	reg-full	44.34	2.5897
Welch	reg-censor	0	5.1374
FFT	reg-censor	0	5.8631
Welch	fooof1	0	7.1895
Welch	fooof3	0	7.8100
FFT	fooof1	0	9.0000
FFT	fooof3	0	10.000

Table S9. **Offset, Positive Slopes Removed,** Odd-Even Reliability - Stop Signal Dataset – Bootstrap Analyses Results

Spectral Decomposition	Aperiodic Estimation	Probability of being the Best	Mean Rank (smaller is better)
Auditory-Auditory			
Welch	fooof0	100	1.5000
Welch	reg-full	100	1.5000
Welch	reg-censor	0.00	3.7902
FFT	fooof0	0.00	3.9405
FFT	reg-full	0.00	4.2693
Welch	fooof1	0.00	6.6290
FFT	Rec-censor	0.00	7.0399
Welch	fooof3	0.00	7.3311
FFT	fooof1	0.00	9.0000
FFT	fooof3	0.00	10.000
Auditory-Visual			
Welch	fooof0	96.10	1.5657
Welch	reg-full	96.10	1.5657
FFT	fooof0	3.98	2.9686
FFT	reg-full	0.20	3.9247
Welch	reg-censor	0.00	4.9753
WElch	fooof1	0.00	6.3130
Welch	fooof3	0.00	6.8594
FFT	reg-censor	0.00	7.8276
FFT	fooof1	0.00	9.0000
FFT	fooof3	0.00	10.000
Visual-Auditory			
Welch	fooof0	100	1.5000
Welch	reg-full	100	1.5000
FFT	fooof0	0.00	3.4942
FFT	reg-full	0.00	3.5061
Welch	reg-censor	0.00	4.9997
FFT	reg-censor	0.00	6.1370
Welch	fooof1	0.00	7.3497
Welch	fooof3	0.00	7.5133
FFT	fooof1	0.00	9.0000
FFT	fooof3	0.00	10.000
Visual-Visual			
Welch	fooof0	87.32.	1.7298
Welch	reg-full	87.23	1.7298
FFT	reg-full	10.62	2.8987
FFT	fooof0	3.74	3.6417
Welch	reg-censor	0.00	5.0335
FFT	reg-censor	0.00	5.9669
Welch	fooof3	0.00	7.3039
Welch	fooof1	0.00	7.6957
FFT	fooof1	0.00	9.0000
FFT	fooof3	0.00	10.000

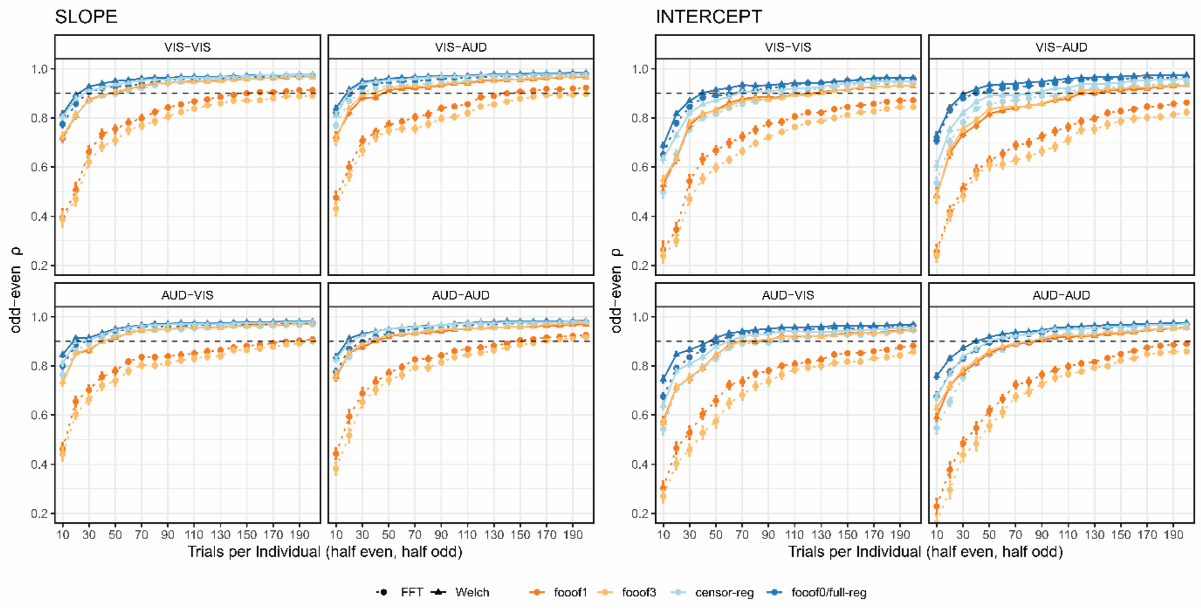

Figure S5. Internal consistency (odd-even reliability) as a function of trial count after removing positive slopes for slope (left) and intercept (right) across aperiodic estimation methods, shown separately for each version of the stop-signal task. The dashed horizontal line represents a reliability threshold of .90, considered acceptable.

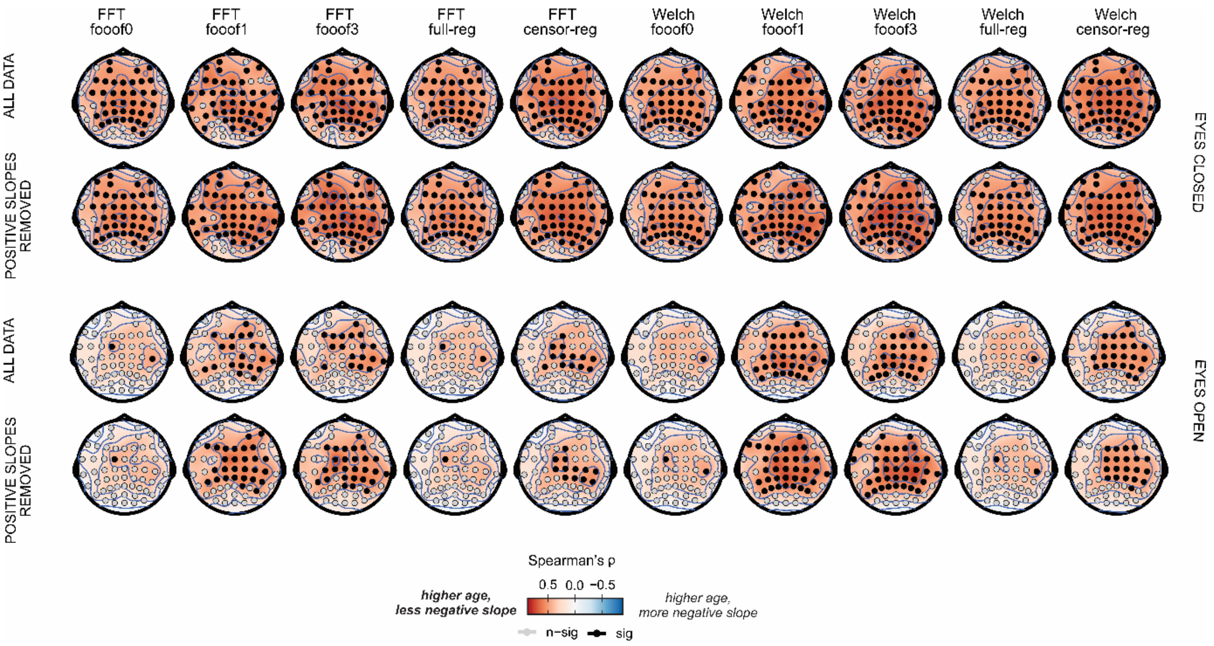

Figure S6. Electrode-Level Correlations Between Age and Slope in the Resting-State Dataset. Spearman's rho correlations between age and slope at each electrode across aperiodic estimation methods for eyes-closed (top) and eyes-open (bottom) resting-state conditions, shown for the full slope dataset (upper) and after removing positive slopes (lower). Statistical significance (p < .05) was assessed using a permutation test. *FFT*, fast Fourier transform; *Welch*, Welch's method; *fooof0, fooof1, and fooof3*, *fooof*-derived slope estimates with the corresponding number of modeled peaks; *full-reg*, full regression approach; *censor-reg*, censored regression approach.

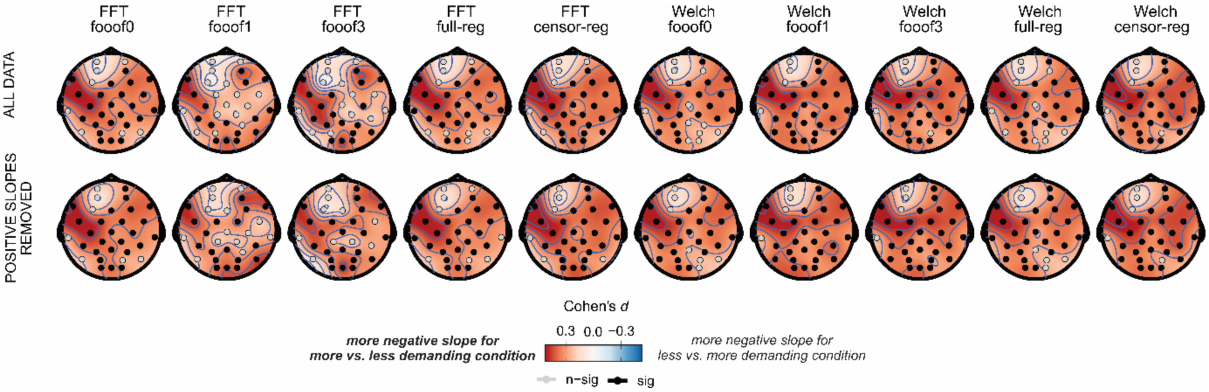

Figure S7. Electrode-Level Experimental Effects in the Stop-Signal Dataset. Effect sizes (Cohen's *d*) for slope comparisons between AUD-VIS and VIS-VIS at each electrode across aperiodic estimation methods, presented for the full slope dataset (top) and after removing positive slopes (bottom). Positive values indicate a more negative slope for AUD-VIS relative to VIS-VIS. Statistical significance ($p < .05$) was assessed using a permutation test.
